# Longitudinal patterns of leukoaraiosis and brain atrophy in symptomatic small vessel disease

**DOI:** 10.1093/brain/aww009

**Published:** 2016-03-01

**Authors:** Christian Lambert, Philip Benjamin, Eva Zeestraten, Andrew J. Lawrence, Thomas R. Barrick, Hugh S. Markus

**Affiliations:** ^1^ Neurosciences Research Centre, Cardiovascular and Cell Sciences Research Institute, St George’s University of London, SW17 0RE, UK; ^2^ Department of Radiology, Charing Cross Campus, Imperial College NHS Trust, London W6 8RP, UK; ^3^ Stroke Research Group, Division of Clinical Neurosciences, University of Cambridge, CB2 0QQ, UK

**Keywords:** small vessel disease, atrophy, longitudinal, voxel-based quantification, white matter hyperintensities

## Abstract

Cerebral small vessel disease is a common condition associated with lacunar stroke, cognitive impairment and significant functional morbidity. White matter hyperintensities and brain atrophy, seen on magnetic resonance imaging, are correlated with increasing disease severity. However, how the two are related remains an open question. To better define the relationship between white matter hyperintensity growth and brain atrophy, we applied a semi-automated magnetic resonance imaging segmentation analysis pipeline to a 3-year longitudinal cohort of 99 subjects with symptomatic small vessel disease, who were followed-up for ≥1 years. Using a novel two-stage warping pipeline with tissue repair step, voxel-by-voxel rate of change maps were calculated for each tissue class (grey matter, white matter, white matter hyperintensities and lacunes) for each individual. These maps capture both the distribution of disease and spatial information showing local rates of growth and atrophy. These were analysed to answer three primary questions: first, is there a relationship between whole brain atrophy and magnetic resonance imaging markers of small vessel disease (white matter hyperintensities or lacune volume)? Second, is there regional variation within the cerebral white matter in the rate of white matter hyperintensity progression? Finally, are there regionally specific relationships between the rates of white matter hyperintensity progression and cortical grey matter atrophy? We demonstrate that the rates of white matter hyperintensity expansion and grey matter atrophy are strongly correlated (Pearson’s R = −0.69, *P* < 1 × 10 ^−7^ ), and significant grey matter loss and whole brain atrophy occurs annually ( *P* < 0.05). Additionally, the rate of white matter hyperintensity growth was heterogeneous, occurring more rapidly within long association fasciculi. Using voxel-based quantification (family-wise error corrected *P* < 0.05), we show the rate of white matter hyperintensity progression is associated with increases in cortical grey matter atrophy rates, in the medial-frontal, orbito-frontal, parietal and occipital regions. Conversely, increased rates of global grey matter atrophy are significantly associated with faster white matter hyperintensity growth in the frontal and parietal regions. Together, these results link the progression of white matter hyperintensities with increasing rates of regional grey matter atrophy, and demonstrate that grey matter atrophy is the major contributor to whole brain atrophy in symptomatic cerebral small vessel disease. These measures provide novel insights into the longitudinal pathogenesis of small vessel disease, and imply that therapies aimed at reducing progression of white matter hyperintensities via end-arteriole damage may protect against secondary brain atrophy and consequent functional morbidity.

## Teaser

The relationship between white matter hyperintensity (WMH) progression and brain atrophy in small vessel disease is unclear. In a longitudinal study, Lambert *et al.* show that WMH progression occurs more rapidly within the long association fasciculi, and is associated with accelerated cortical atrophy. Preventing WMH progression may reduce secondary degeneration.

## Graphical abstract

**Figure:**
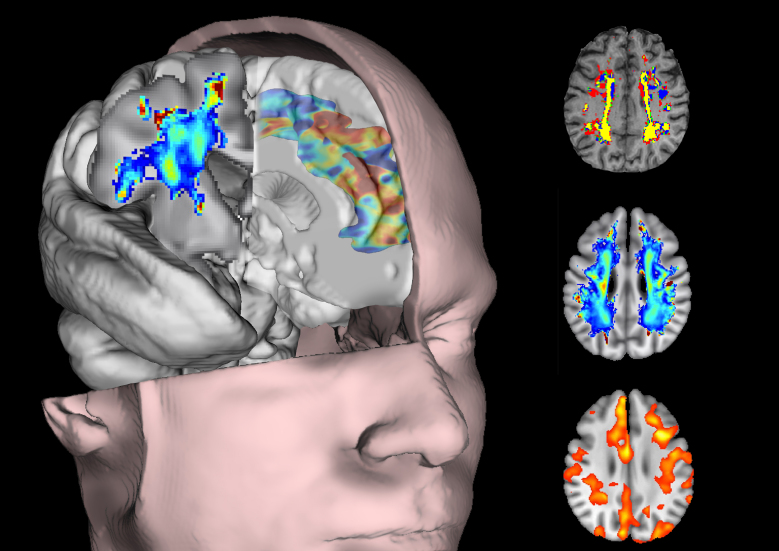
The relationship between white matter hyperintensity (WMH) progression and brain atrophy in small vessel disease is unclear. In a longitudinal study, Lambert *et al.* show that WMH progression occurs more rapidly within the long association fasciculi, and is associated with accelerated cortical atrophy. Preventing WMH progression may reduce secondary degeneration.

## Introduction

Cerebral small vessel disease is a group of heterogeneous disorders that affect the small vessels of the brain ( Pantoni, 2010 ). Small vessel disease is characterized by typical radiological changes on MRI including white matter hyperintensities (WMH), lacunar infarcts, cerebral microbleeds and brain atrophy ( Gouw *et al.* , 2011 ), and is associated with vascular risk factors, particularly hypertension ( Jeerakathil *et al.* , 2004 ). Small vessel disease is a highly prevalent disease that increases with age ( De Leeuw *et al.* , 2001 ), and is part of a clinical spectrum that ranges from asymptomatic disease detected on brain imaging in healthy adults through to extensive WMH and lacunar infarct in vascular dementia ( Pantoni, 2010 ; Patel and Markus, 2011 ).

WMHs, best observed on T _2_ -weighted magnetic resonance images, are one of the conventional MRI markers of small vessel disease ( Gouw *et al.* , 2011 ). However, *ex vivo* correlates of these high signal regions reveal a spectrum of pathophysiological processes including white matter demyelination and rarefaction through to axonal loss and gliosis ( Braffman *et al.* , 1988 ; Munoz *et al.* , 1993 ; Gouw *et al.* , 2011 ). Many of these changes are thought to have an ischaemic pathogenesis ( Fernando *et al.* , 2006 ). The arterial supply to deep white matter regions is via long penetrating arteries that originate at the pial surface, and then travel up to 5 cm with myelinated axons to irrigate deep white matter structures ( Pantoni and Garcia, 1997 ). These end arterial regions form watershed areas that are particularly prone to ischaemic damage in response to changes in cerebral blood flow. Functionally, the rate of WMH progression is associated with decline in verbal IQ ( Garde *et al.* , 2005 ) and executive function ( Debette and Markus, 2010 ), and is a predictor of future stroke ( Yamauchi *et al.* , 2002 ) and non-Alzheimer’s dementia ( Steffens *et al.* , 2007 ) risk. Consequently, WMH volume has also been proposed as a clinically relevant disease marker that can be pharmacologically modulated, and has been proposed as an end point in clinical trials ( Dufouil *et al.* , 2005 ).

Previous studies have demonstrated that increasing small vessel disease is associated with increases in both WMH volume and brain atrophy ( Godin *et al.* , 2009 ; Nitkunan *et al.* , 2011 ; Patel and Markus, 2011 ). In particular, the brain atrophy predominantly affects the cortical grey matter ( Du *et al.* , 2005 ) and has been shown to correlate with cognitive impairment ( Nitkunan *et al.* 2011 ; Jokinen *et al.* , 2012 ) and gait decline ( de Laat *et al.* , 2012 ). However, whether the observed vascular lesions independently cause secondary brain atrophy, and if so how this process is mediated, remain open questions ( Appelman *et al.* , 2009 ; Wardlaw *et al.* , 2013 ). Several possibilities have been proposed: it maybe that the brain atrophy is due to white matter damage leading either to subsequent downstream denervation ( O’Sullivan *et al.* , 2001 ), or a result of volumetric loss of white matter constituents such as myelin, axons, and oligodendrocytes ( Appelman *et al.* , 2009 ). Alternatively, intrinsic cortical disease due to processes such as cortical microinfarcts ( Smith *et al.* , 2012 ) may cause cortical grey matter atrophy. Furthermore data on regional variation in the longitudinal rates of atrophy related to rate of WMH progression are lacking, casting uncertainty on the nature and direction of the association between WMH and brain atrophy ( Appelman *et al.* , 2009 ). If a ‘disconnection phenomenon' due to white matter denervation does play a role in brain atrophy, one might expect a relationship between the rates of WMH progression and regional cortical grey matter atrophy. In addition, white matter lesions are known to be spatially heterogenous ( Artero *et al.* , 2004 ; DeCarli *et al.* , 2005 ), progressing at different rates in different cortical locations. Precise mapping of the longitudinal rate of WMH growth would be expected to provide further clues into the underlying pathophysiological process.

To better define the relationship between WMH growth and brain atrophy we developed a 3D spatial model of WMH progression in cerebral small vessel disease, and generated WMH rate maps to show the rate of WMH expansion within each voxel. We used these to characterize the spatial progression of small vessel disease WMH in a prospective longitudinal cohort of individuals with symptomatic small vessel disease (defined as having suffered a prior clinical lacunar syndrome), and determine its association with brain atrophy using whole brain, semi-automated, morphometric techniques. First, we aimed to demonstrate the relationship between the rates of WMH growth and brain atrophy. Secondly, previous studies using region of interest based analysis have shown that the rate of WMH accumulation is non-uniform and more rapid in the fronto-parietal regions. Using the 3D voxel-by-voxel rate maps we define the characteristic pattern of WMH accumulation, and use statistical analysis to identify regions of WMH growth that are correlated with the rate of brain atrophy.

## Materials and methods

### Subjects

Patients in the SCANS (St George’s Cognition and Neuroimaging in Stroke) cohort were studied. This prospective longitudinal study recruited 121 subjects with symptomatic small vessel disease and followed them up longitudinally. Recruitment was from acute stroke units or outpatient stroke clinics in three hospitals covering a contiguous catchment area in South London (St George’s, King’s College and St Thomas’ Hospitals). Inclusion criteria comprised a clinical lacunar syndrome ( Bamford *et al.* , 1987 ) with an anatomically corresponding lacunar infarct in addition to evidence of confluent leukoaraiosis (modified Fazekas grade ≥ 2; Fazekas *et al.* , 1993 ; Hassan *et al.* , 2003 ) on MRI. Exclusion criteria were: any cause of stroke mechanism other than small vessel disease, other major CNS disorders, major psychiatric disorders, any other cause of white matter disease, contraindications to MRI, or non-fluent in English. Patients were studied at least 3 months after their last stroke. All subjects provided written consent, and the study was approved by the local ethics committee.

**Figure 1:**
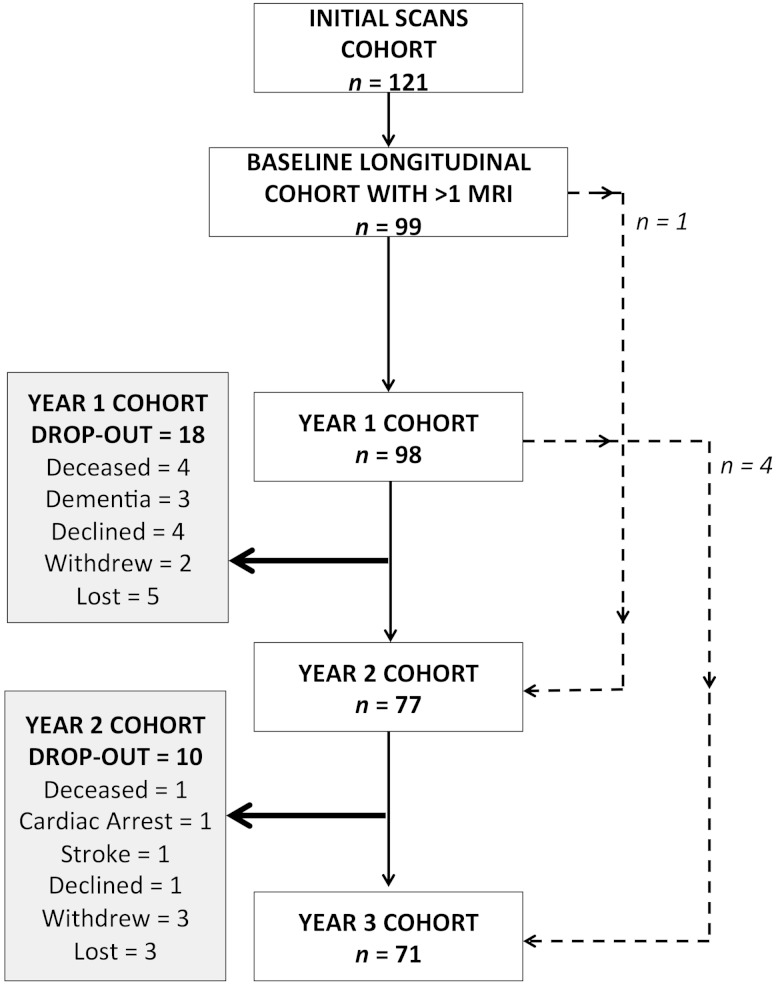
**Longitudinal cohort flow chart.** Flow chart demonstrating the changes in annual cohorts and reasons for subject dropout at each time point. Dashed lines: one subject attended the baseline, missed the Year 1 scan but attended Year 2 onwards. Four subjects missed their Year 2 follow-up but attended all other time points (baseline, Years 1 and 3).

Subjects were invited annually for repeated cognitive testing and MRI scanning. Recruitment began in December 2007 and ended in August 2010. MRI scanning began in January 2008 and was completed in October 2013. In this analysis follow-up data to 3 years were used, providing a maximum of four datasets per individual. Of these, 99 subjects returned at one or more time-points: 98 at Year 1, 77 at Year 2 and 71 at Year 3. One subject attended the baseline and missed the Year 1 follow-up, but attended all subsequent sessions. Four subjects missed the Year 2 follow-up, but subsequently attended at Year 3. These are summarized with the reasons for dropout in Fig. 1 , and a detailed breakdown of the demographics is provided in Table 1 . Additionally, over the period reported there were three new clinical strokes, two lacunar and one cortical haemorrhage. Intercerebral haemorrhage was a predefined end-point and the patient was withdrawn from the study. The two lacunar stroke cases were allowed to remain in the study, as stated in the protocol, although one subsequently withdrew due to disability. All available longitudinal data ( *n* = 99) were used for analysis.

**Table 1:** Summary of cohort demographics at each time point

	**Time point**				
	**Baseline**	**Year 1**	**Year 2**	**Year 3**	**Average**
*n*	99	98	77	71	-
Age at time point (years)	68.9 ( *9.98* )	69.97 ( *10.14* )	70.71 ( *9.24* )	71.71 ( *9.24* )	70.17 ( *9.70* )
Female	33 (33%)	33 (34%)	26 (34%)	26 (37%)	30 (34%)
Male	66 (67%)	65 (66%)	51 (66%)	45 (63%)	57 (66%)
Hypertension	92 (93%)	91 (93%)	71 (92%)	66 (93%)	80 (93%)
Hypercholesterolaemia	85 (86%)	84 (86%)	65 (84%)	62 (87%)	74 (86%)
Current smoker	21 (21%)	21 (21%)	16 (21%)	15 (21%)	18 (21%)
Ex-smoker	34 (34%)	33 (34%)	25 (33%)	22 (31%)	29 (33%)
Diabetes	19 (19%)	19 (19%)	18 (23%)	17 (24%)	18 (21%)

### Image acquisition

All magnetic resonance images were acquired using the Signa HD 1.5 T Magnetic Resonance Scanner (General Electric) at St George’s, University of London. The maximum gradient amplitude was 33 mTm ^−1^ and a proprietary 8-channel head coil was used. All subjects were placed in the head coil and an alignment marker was used at the nasal bridge. Velcro straps and foam pads were used where possible to minimize head movement. Whole brain T _1_ -weighted and fluid-attenuated inversion recovery (FLAIR) images were acquired for each subject using the following protocol: (i) FLAIR sequence: repetition time/echo time/inversion time = 9000/130/2200 ms, field of view = 240 × 240 mm ^2^ , matrix = 256 × 192, 28 axial slices of 5-mm thickness providing a final image resolution of 0.47 × 0.47 × 5 mm; and (ii) spoiled gradient echo recalled T _1_ -weighted (SPGR) 3D coronal sequence: repetition time/echo time = 11.5/5 ms, field of view = 240 × 240 mm ^2^ , matrix = 256 × 192, flip angle = 18°, 176 coronal slices of 1.1-mm thickness providing a final image isotropic resolution of 1.1 mm.

### Preprocessing

Initially the raw DICOMs were imported using Statistical Parametric Mapping 8 (SPM8) software ( http://www.fil.ion.ucl.ac.uk/spm/software/spm8 /), and each image checked to ensure a common orientation. The T _1_ -weighted and FLAIR images were aligned using an affine co-registration in SPM for each individual, before being co-registered to MNI orientation and reslicing to 1 mm isotropic voxel resolution in subject space using fourth degree b-spline interpolation.

### Definitions

The longitudinal pipeline involved working principally in four separate image spaces and exploited a two step-warping procedure to characterize the temporal changes. For reference, the main terminology and space definitions have been provided in Supplementary Fig. 1 .

### Segmentation

**Figure 2:**
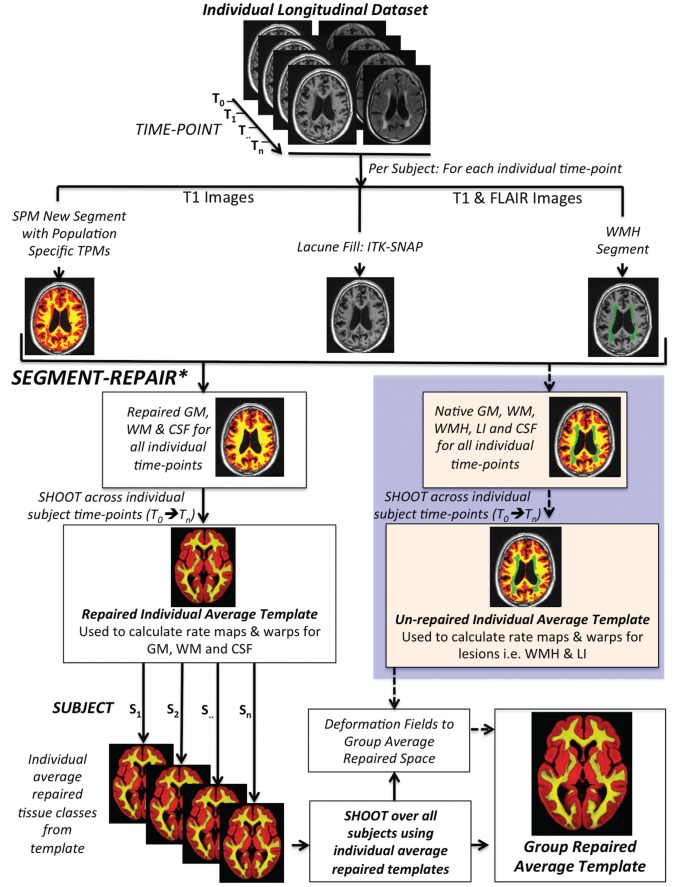
**Summary of the longitudinal preprocessing pipeline.** *The tissue repair step is outlined in the methods and detailed further in Lambert *et al.* (2015) . GM = grey matter; WM = white matter; LI = lacunar infarct.

The segmentation steps were adapted and optimized to our study population as described in Lambert *et al.* (2015) , but are fully summarized below for reference. In essence, the adapted segmentation pipeline consisted of four steps. First, a group average template was created and the T _1_ -weighted and FLAIR images were warped to this space. Second, the warped T _1_ -weighted and FLAIR images were used to create population-specific tissue probability maps (TPMs). Third, the new TPMs were used to resegment the native images creating grey matter, white matter, CSF and WMH tissue classes. These were then combined with the manually defined lacune regions of interest, resulting in five tissue classes per individual. Finally, a tissue repair step was performed to generate repaired grey matter, white matter and CSF maps for each individual dataset. Description of the subsequent longitudinal warping procedure that uses all the segmented tissue maps will be detailed separately after the segmentation steps. Figure 2 summarizes the principle segmentation steps (two and three detailed above) and the subsequent longitudinal two stage warping procedure. Technical details for the generation of population-specific TPMs may be found in Lambert *et al.* , (2013) . Additional details for each step have been provided in the Supplementary material . For clarity, SPM8 New Segment was used to develop and apply the segmentation pipeline (as this version was available during initial methodological development) but with customized TPMs generated from our data rather than default SPM TPMs. SPM12 was available for our later analysis and was used for all subsequent longitudinal warping (via the new ‘Shoot’ toolbox) and statistical analysis.

#### Step 1: Create initial group average space

Initially the native space T _1_ -weighted images were segmented into grey matter, white matter and CSF tissue classes using the unified segmentation (‘New Segment’) within SPM8 ( Ashburner and Friston, 2005 ). These segmentations were registered to a common group-average 1 mm isotropic voxel template image using a diffeomorphic registration algorithm ( Ashburner and Friston, 2011 ), and the T _1_ -weighted and FLAIR images warped to this space. These images were skull-stripped using the warped segmentations at a tissue probability threshold of 0.1, and manually checked and corrected where necessary.

#### Step 2: Create population-specific tissue probability maps

Population-specific TPMs were created for three tissue classes (grey matter, white matter and CSF) using the warped, skull-stripped T _1_ -weighted images and a Modified Mixture of Gaussians algorithm to account for anatomical features specific to the study cohort, such as larger ventricles. Using the warped skull-stripped FLAIR and T _1_ -weighted images, TPMs were generated for the WMHs using a Modified Multivariate Mixture of Gaussians ( Lambert *et al.* , 2013 ). These TPMs then replaced the default maps supplied by SPM in the New Segment pipeline.

#### Step 3: Re-segmenting the native space images

The new TPMs were used to resegment the native space structural images into grey matter, white matter, CSF and WMH. The central corpus callosum and fornix were excluded from the WMH segmentation by masking with a group average mask. This was performed to reduce the regional variation of voxel intensities in these structures that cause frequent tissue misclassification. The WMH segmentations were binarized at a manually determined threshold for each individual. This threshold was determined by overlaying the segmentations onto the corresponding FLAIR images using ITK-SNAP version 2.2.0 ( www.itksnap.org ) ( Yushkevich *et al.* , 2006 ), to ensure each individual WMH map accurately defined the spatial extent of the lesions. This step was performed independently by two raters (C.L. and E.Z.), and inter- and intra-rater reliability metrics checked using 10 randomly selected scans across all time points, duplicated to provide a total set of 20 scans per rater, each set randomly reordered for each rater to avoid any sequence bias. Both raters were blinded with respect to subject and time-point of each scan. Each binary map was further checked and corrected where necessary using ITK-SNAP. The WMH manual refinement removed areas of mis-segmentation that were apparent in some areas with similar voxel intensity profiles to WMHs, and was most often required in the hippocampus and posterior insula. This step did not alter the contours or extent of the automatically delineated WMH lesions. Areas of lacunar infarction were manually identified on native space T _1_ -weighted images using published criteria ( Benjamin *et al.* , 2014 ). This was aided by overlaying CSF segmentations on corresponding FLAIR images to identify regions of misclassification. Regions were segmented using a space-filling algorithm in ITK-SNAP, using voxel intensity thresholds (set to between 350 and 500) with 500 iterations. These results were visually checked and manually refined where necessary. This step was performed independently by two raters (C.L. and P.B.), and inter- and intra-rater reliability checked using 20 randomly selected scans across all time points, as detailed above. Again the raters were blinded to the subject and time-point of each scan.

#### Step 4: Tissue repair

One of the problems associated with segmenting brains with WMH and lacunar infarct is that regions of damage can be misclassified into erroneous tissue types due to changing tissue intensity profiles as a result of pathology. Regions of WMH are often classed as grey matter, and gliosis within lacunar infarct as CSF, in a standard segmentation result from SPM8. This misclassification will cause inaccuracies when warping the segmentations together to a group average template, and impair the ability to accurately map areas of damage to a common space. For example, a region of gliosis caused by a lacune in the head of the caudate would be warped with the CSF tissue class into the adjacent lateral ventricle. To overcome this limitation a segmentation repair step was undertaken. The WMH binary maps were used to identify brain regions that should be classed as white matter. These regions were subsequently corrected in the corresponding white and grey matter segmentations, by zeroing the WMH regions in grey matter, and adding them to white matter. As lacunar infarct may cross tissue boundaries they were mapped to the initial group average space and the TPMs were used to define the most likely tissue class in the lacunar infarct regions over all time-points. This was then used to reclassify the native grey matter, white matter and CSF maps.

To further improve the segmentations, each baseline T _1_ -weighted image was processed using the recon-all pipeline in Freesurfer (version 5.0; Fischl *et al.* , 2002 ). The resultant parcellation image was binarized and combined with the repaired segmentations to create an individual brain mask. Each mask was then manually checked in ITK-SNAP to remove any erroneously classified brain voxels around the margins of the skull and dura. Copies of individual baseline brain masks were then co-registered using SPM12 to all future time points using the corresponding T _1_ -weighted images. The final brain mask for each time point was then used to remove any erroneously classified grey and white matter voxels in the segmentations and these were included in the CSF images. At each time point, the final result was visualized and, if necessary, the masks were further refined manually.

For each scan, the unrepaired segmentations were used to calculate tissue volumes. The WMH, lacunar infarct, total cerebral volume (defined as the sum of grey matter, white matter and WMH at a tissue probability threshold ≥ 0.2) and total intracranial volume (defined as total cerebral volume plus the CSF volume at a set tissue probability threshold ≥ 0.2, constrained by the final brain inclusion mask) were calculated in mm ^3^ .

Reliability metrics were calculated as per Dunn *et al.* (2013) . The inter-rater reliability metrics were calculated using the mean values for each rater. Good measures of reliability were obtained for the MRI measures that needed manual refinement. For WMHs, the inter-rater reliability metrics were: standard error of mean (SEM) = 247 mm ^3^ , mean variability = 7.78% [standard deviation (SD) = 3.03%], Pearson’s intra-class correlation coefficient = 0.98. The intra-rater reliability metrics were: SEM = 187 mm ^3^ , mean variability = 5.57% (SD = 3.99%), Pearson’s intraclass correlation coefficient = 0.99. For lacunar infarct, the inter-rater reliability metrics were: SEM = 3 mm ^3^ , mean variability = 7.93% (SD = 4.89%), Pearson’s intra-class correlation coefficient = 0.99. The corresponding intra-rater reliability metrics were: SEM = 2 mm ^3^ , mean variability = 4.32% (SD = 4.19%), Pearson’s intraclass correlation coefficient = 0.99. There were no significant differences between the volumes measured by the two raters.

### Longitudinal warping pipeline

To calculate longitudinal volumetric changes in small vessel disease and to enable statistical analysis regardless of the number of time points, we developed a two-stage warping pipeline by adapting the available SPM framework ( Fig. 2 ) that first warped each subject to an individual average template that represents the average mid-point brain. This step provides a voxel-wise trajectory, based on the non-linear deformations to the average individual brain, for each time point. These are expressed as divergence maps (for each time point) in SPM as detailed below. To create a ‘rate map’ we performed a voxel-wise linear fit between the divergence maps, using the time between scans as the basis ( Ashburner and Ridgway, 2012 ). These individual rate maps show the relative speed of tissue expansion or contraction for every voxel in the brain per year and were used for analysis.

#### Step 1: Individual average space

Initially for each individual, the five tissue type maps (grey matter, white matter, CSF, WMH and lacunar infarct), and all available time points (between two and four per subject) were warped to an individual average template using the Shoot toolbox in SPM12. This produced Jacobian determinant, warp and velocity fields for each time-point in addition to the individual average tissue template images. The velocity fields were used to calculate divergence maps using the in-built SPM12 function spm_shoot_divergence. For each voxel, the divergence maps quantify the diffeomorphic distance between the subject time point image from the average image, and represent the amount of expansion or contraction required to warp it to the average image. For each voxel, across all divergence maps for an individual, a linear fit was calculated with respect to the time between scans to generate a single ‘rate map’ for each subject. These steps were then repeated using the three repaired tissue classes to produce a repaired individual average image and a rate map for each corrected tissue type.

#### Step 2: Group average space

Individual repaired average tissue class templates were generated by the longitudinal warping in Step 1. In Step 2, the three average tissue classes for each individual were used in the Shoot pipeline to provide deformations to a group average space.

### Longitudinal analysis

For each subject, the following images from individual average space were warped to the group average template: grey matter template, white matter template, WMH template, lesion rate maps and repaired rate maps. Each of the warped tissue classes was multiplied by the warped rate maps (WMH by lesioned-rate images, grey matter and white matter by repaired-rate images). These unsmoothed images were used to produce the group average rate images for each tissue class, reflecting the average expansion and atrophy across the whole brain in the entire cohort. Because the distribution of WMH was variable over the cohort, a normalized average WMH rate map was also calculated by dividing the sum of all the warped WMH rates by the sum of all warped binarized WMH maps i.e. for each voxel only data from individuals where a specific voxel contains a lesion, rather than over the entire group, were used to generate an average for that point.

To statistically analyse the tissue-specific rate maps, a voxel-based quantification approach was adopted ( Draganski *et al.* , 2011 ). Specifically the rate maps for WMH and repaired grey matter were treated as parameter maps, and the combined weighting/smoothing procedure described in Draganski *et al.* (2011) used to produce warp-weighted average images. These avoid the parameter value changes caused by the necessary Gaussian smoothing in standardized space and have previously been used in a similar fashion for cortical thickness parameter maps ( Hutton *et al.* , 2009 ). In this work, the grey matter and WMH warp-weighted average rate maps were smoothed using a 6 mm full-width at half-maximum Gaussian kernel.

### Whole brain volumetric MRI parameters

Total cerebral volume, grey matter, white matter, lacunar infarct, and WMH volumes were calculated for each individual and used to calculate percentage change from baseline and annualized rate of volumetric change. For each MRI parameter annualized rate of change was calculated from all available time points (e.g. time in years from baseline) using a least squares linear fit in MATLAB 2013a. These were calculated both using each time point cohort (i.e. only taking data from baseline to the corresponding time-point) to assess how these measures changed as the cohort declined due to dropout, and also using the entire population over all available time points to generate a single summative measure.

### Statistical data analysis

All analyses were performed in MATLAB 2013a unless specified, and the data were grouped either according to time-point or outcome as specified. Tests between time points used a one-tailed test given the *a priori* expectation that brain volumes decline and lesioned tissue increases with time, whereas all other *t* -tests were two-tailed. Data distributions were assessed using histograms. The normally distributed data were explored initially using an ANOVA, and if significant a two-sample *t* -test was used to examine differences between groups. Non-parametric data were tested using a Kruskal-Wallis test. Pearson’s correlation was used to calculate correlation coefficients (r). Results significant at *P* < 0.05 were reported.

### Statistical image analysis

All analysis was performed in SPM12. All group analyses were performed in the population optimized group average space. The objective was to identify regions where the rate of grey matter atrophy and WMH growth were significantly correlated with one another. To achieve this, the warp-weighted average rate maps (grey matter and WMH) were analysed using multiple regression models in SPM. The dependent variables were the average whole brain rate values (as specified in the ‘Whole brain volumetric MRI parameters’ section) for either grey matter or WMH. Confounding variables were baseline age, lacunar infarct volume, total intracranial volume and gender. In models using the grey matter rate maps, the baseline WMH volumes were also included as a confounding covariate.

The first multiple regression model tested the grey matter rate maps for positive and negative correlations with average WMH rate and age (four statistical tests total). In the second, the WMH rate maps were tested for positive and negative correlations with average grey matter rate and age (four statistical tests total).

In all statistical analyses the regions that survived family-wise error (FWE) multiple comparison correction at *P* < 0.05 were considered significant. All significant results are displayed at both *P* < 0.001 uncorrected and FWE corrected *P* < 0.05 and the legend labelled accordingly. All images are shown using the neurological convention.

## Results

### Demographics

A total of 99 subjects had more than one time point, and were included in the analysis as outlined in the ‘Materials and methods’ section. The physical longitudinal demographics including vascular risk factors are shown in Table 1 . Functional baseline averaged measures include: Rankin (1.1 ± 1.0), Mini-Mental State Examination (27.9 ± 2.4) and National Adult Reading Test IQ (99.8 ± 15.4). A detailed breakdown of baseline characteristics of this cohort have been described in detail elsewhere ( Lawrence *et al.* , 2013 ).

### MRI measures

#### Overall

The changes in MRI parameters are summarized in Table 2 and Supplementary Fig. 2 . The average total cerebral volume over all available time points was 1020 × 10 ^3^ mm ^3^ (SD = 100 × 10 ^3^ mm ^3^ , range 740–1240 × 10 ^3^ mm ^3^ ), and average volumes of the composite compartments were 649 × 10 ^3^ mm ^3^ for grey matter (SD = 66 × 10 ^3^ mm ^3^ , range 502–779 × 10 ^3^ mm ^3^ ) and 322 × 10 ^3^ mm ^3^ for white matter (SD = 59 × 10 ^3^ mm ^3^ , range 163–471 × 10 ^3^ mm ^3^ ). The median WMH volume was 44.1 × 10 ^3^ mm ^3^ (SD = 29.3 × 10 ^3^ mm ^3^ , range 3.5–150.2 × 10 ^3^ mm ^3^ ). At baseline, WMH volume did not correlate with age (Pearson’s coefficient, r = −0.07, *P* = 0.47) or total brain volume (Pearson’s coefficient, r = 0.13, *P* = 0.14).

**Table 2:** Summary of MRI parameters at each time point

	**Time point**				
	**Baseline**	**Year 1**	**Year 2**	**Year 3**	**Overall**
*n*	99	98	77	71	-
**Demographics and risk factors**					
Age at time point (years)	68.90 (9.98)	69.97 (10.14)	70.71 (9.24)	71.71 (9.24)	70.17 (9.70)
Female ( *n* )	33 (33%)	33 (34%)	26 (34%)	26 (37%)	-
Male ( *n* )	66 (67%)	65 (66%)	51 (66%)	45 (63%)	-
Hypertension ( *n* )	92 (93%)	91 (93%)	71 (92%)	66 (93%)	-
Hypercholesterolaemia ( *n* )	85 (86%)	84 (86%)	65 (84%)	62 (87%)	-
Current smoker ( *n* )	21 (21%)	21 (21%)	16 (21%)	15 (21%)	-
Ex-smoker ( *n* )	34 (34%)	33 (34%)	25 (32%)	22 (31%)	-
Diabetes ( *n* )	19 (19%)	19 (19%)	18 (23%)	17 (24%)	-
**Volume measurements**					
Mean grey matter (mm ^3^ )	669 596 (65 898)	643 210 (64 077)	640 944 (63 226)	635 230 (64 979)	648 634 (65 745)
Mean white matter (mm ^3^ )	331 400 (59 000)	323 568 (56 882)	321 063 (55 211)	311 455 (63 210)	322 763 (58 645)
Mean total cerebral volume (mm ^3^ )	1 038 030 (104 134)	1 012 586 (101 527)	1 008 459 (98 714)	995 645 (110 147)	1 015 480 (104 204)
Median WMH (mm ^3^ )	31 899 (26 640)	37 604 (31 215)	39 071 (29 606)	43 338 (28 400)	44 080 (29 257)
Median lacune (mm ^3^ )	251 (713)	317 (799)	317 (844)	377 (882)	317 (802)
**Percentage change from baseline (%)**					
Mean total grey matter	-	−3.83 (2.00)	−4.45 (1.81)	−5.27 (1.88)	−4.44 (1.99)
Mean total white matter	-	−2.21 (3.93)	−4.50 (4.80)	−7.86 (6.55)	−4.56 (5.56)
Mean total brain	-	−2.39 (1.16)	−3.09 (1.54)	−4.19 (2.27)	−3.13 (1.81)
Median total WMH	-	+24.40 (25.26)	+46.17 (34.20)	+59.55 (54.45)	+38.50 (42.88)
Median lacune	-	+13.43 (21.97)	+22.22 (24.60)	+29.92 (23.15)	+27.75 (27.05)
** Annualized fitted rates (mm ^3^ /year) **					
Mean grey matter	-	−25 647 (13 692)	−14 949 (6314)	−11 021 (4089)	−14 860 (10 866)
Mean white matter	-	−7845 (12 973)	−7591 (7390)	−8506 (6781)	−8838 (7340)
Mean total cerebral volume	-	−24 958 (12 234)	−16 080 (7816)	−13 670 (7525)	−15 873 (9997)
Median WMH	-	+5860 (9043)	+5385 (4502)	+5127 (3880)	+5626 (8095)
Median lacune	-	+37 (129)	+30 (75)	+29 (75)	+30 (89)

SD is shown in brackets unless otherwise stated.

#### Longitudinal

These are summarized in Table 2 and Supplementary Fig. 2 . There was a progressive decline in total cerebral volume, grey matter and white matter over time with annualized rates of −159 × 10 ^3^ mm ^3^ /year, −149.6 × 10 ^3^ mm ^3^ /year and −88 × 10 ^3^ mm ^3^ /year, respectively. There was a significant difference grey matter and total cerebral volume between baseline and Years 1, 2, and 3 ( *P* < 0.005 for grey matter, *P* < 0.05 for total cerebral volume), and between the baseline and final white matter volumes ( *P* < 0.05), which may indicate that the progressive loss in total cerebral volume is being largely contributed by grey matter changes. The rate of total cerebral volume and grey matter decline was reduced significantly in the Year 1 and Year 2 cohorts ( *P* < 1 × 10 ^−4^ ). We found no correlations between the rates of grey matter, white matter and total cerebral volume change compared to their respective baseline volumes.

In contrast, the overall volume of WMH increased progressively each year. The median annualized WMH growth rate was 5.6 × 10 ^3^ mm ^3^ /year (SD = 8.1 × 10 ^3^ mm ^3^ , range 0.82–65.9 × 10 ^3^ mm ^3^ /year). There was a significant positive correlation between total baseline WMH volume and the annualized rate of WMH growth (Pearson’s R = 0.49, *P* < 1 × 10 ^−4^ ). Examining the separate tissue types, the rate of WMH expansion was strongly correlated with rate of grey matter atrophy (Pearson’s R = −0.69, *P* < 1 × 10 ^−7^ ) and weakly correlated with white matter (Pearson’s R = −0.26, *P* = 0.01). Additionally, the median annualized rates (mm ^3^ /year) for WMH and lacunar infarct were strongly positively correlated (Pearson’s R = 0.59, *P* < 1 × 10 ^−7^ ).

Finally the median rate of total lacunar infarct volume increase remained reasonably static over all time points (30 mm ^3^ /year). This parameter was moderately negatively correlated with grey matter (Pearson’s R = 0.34, *P* < 0.005), but not white matter or total cerebral volume.

### Average whole brain white matter hyperintensity changes

**Figure 3:**
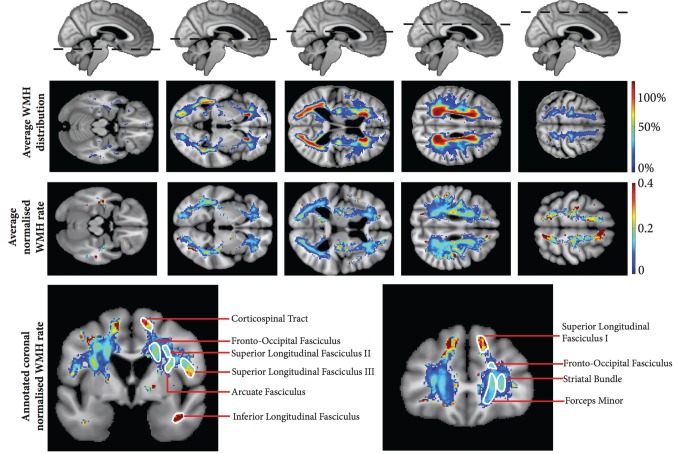
**Group average WMH rate maps.** Axial projections of group average WMH rate maps shown using the neurological convention. Top row shows overall average over the group, bottom row has been normalized by averaging across individual subject voxels only where WMH is present and represents the average rate if WMH is found in a particular voxel. Bottom images display two coronal sections with annotation of the corresponding white matter anatomy according to Schmahmann’s white matter atlas ( Schmahmann and Pandya, 2009 ).

Figure 3 shows the group average distribution of WMH, and normalized WMH rate. The normalized map represents the average rate of growth in subjects where a lesion is located in a particular voxel, whereas the WMH distribution map provides complementary information regarding the actual probability of a lesion being present in a specific voxel across the population. Overall the rate of WMH growth was greater in the gyral white matter close to the cortical mantle, at the edge of the WMH distributions in regions of low probability WMH lesions. This is to be expected, as the WMH lesion edges in subjects with severe, confluent high volume disease would be located in these gyral regions, and is consistent with the earlier observed positive correlation between WMH volume and WMH growth rate. This effect was most apparent in the fronto-parieto-occipital regions. However, there was also heterogeneity in the rate of WMH growth in the deeper non-gyral white matter. While this was highest in the region of the corticospinal tract and parietal centrum semiovale, regions of faster growth were observed to conform to major white matter fasciculi when compared against a white matter atlas ( Schmahmann and Pandya, 2009 ). These are annotated in Fig. 3 , and include the superior longitudinal fasciculus, fronto-occipital fasciculus, arcuate fasciculus, inferior longitudinal fasciculus, forceps major and minor and the striatal bundle. This indicates that development of WMH changes occur more rapidly within major white matter fasciculi, particularly long association white matter bundles. This observation crosses vascular boundaries ( Fig. 3 ) and is therefore not associated with larger arterial changes or cortical microinfarcts but instead the end arteriole supply. If the former were responsible, the spatial pattern of WMH rate would be expected to show acceleration along the supplying perforators.

### Grey matter rate maps

**Figure 4:**
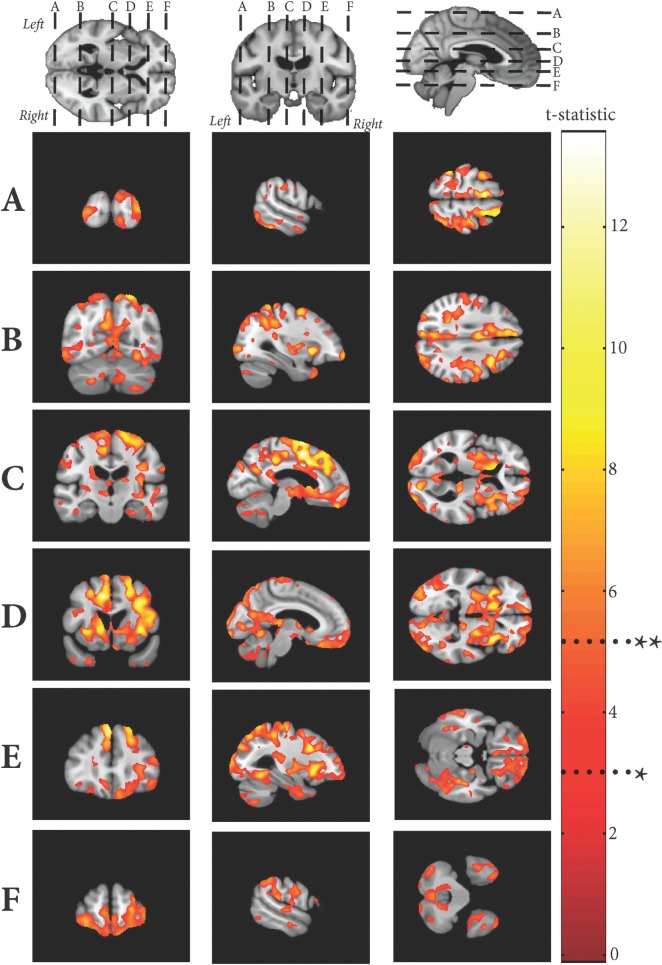
**Significant regions of negative correlation between grey matter rate maps and overall WMH rate** . T-statistic shown at * *P* < 0.001 uncorrected and FWE ^**^P < 0.05.

Regions of the grey matter volumetric rate of change maps that were significantly negatively correlated with WMH growth rate while controlling for baseline age, gender, baseline WMH volume, lacunar infarct volume and total intracranial volume are shown in Fig. 4 . There was no significant positive correlation between grey matter and WMH rates, and there was no significant association between grey matter rates and age in our cohort. Significant results show increased rates of grey matter atrophy related to increasing WMH growth in the occipital lobe, superior parietal lobe (including the precuneus), prefrontal and orbitofrontal regions, in addition to the striatum. Additionally, there were symmetrical changes observed in the cerebellum and dentate nuclei. There was notable sparing of the medial temporal lobe and hippocampus.

### White matter hyperintensity rate maps

**Figure 5:**
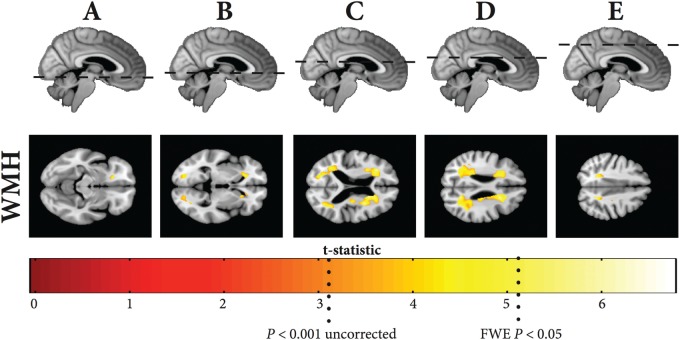
**Significant regions of negative correlation between WMH rate maps and with overall grey matter rate** . T-statistic shown at * *P* < 0.001 uncorrected and FWE ^**^P < 0.05.

Regions of the WMH rate maps that were significantly negatively correlated with grey matter rate of change whilst controlling for baseline age, gender, baseline lacunar infarct volume and total intracranial volume are shown in Fig. 5 . There was no significant positive correlation, and there was no significant association between WMH growth rate with age in our cohort. Significant results show that regions where the rate of WMH expansion was most significantly correlated with increasing grey matter atrophy were in the parietal/precuneus region around the centrum semiovale, in the occipital lobe in the optic radiations and forceps major, and frontally adjacent to the forceps minor.

### Dropout cohort

Because of significant changes in the measured volumes compared to the baseline but non-significant changes between the later time points, a subanalysis was performed to examine whether the characteristics of the subjects who withdrew from the study could account for this. The results of this analysis are summarized in Table 3 . To note, the apparent differences in the demographic and risk factor columns between Tables 1 and 3 are caused by five subjects who missed a time point before reattending (as shown in Fig. 1 ). This slightly modifies the cross-sectional results shown in Table 3 , which summarizes results obtained for all subjects who continue in the study, versus those who dropout. Dropouts had significantly higher burdens of small vessel disease as reflected by WMH volume at both time points. Furthermore, the annualized rate of expansion for WMH was significantly higher in drop-outs for these variables at both time points compared to participants who continued in the study. The differences between median WMH volumes and annualized WMH rate between the Year 1 and 2 dropout groups were not significant. The reasons for subject dropout are provided in Supplementary Table 1 .

**Table 3:** Summary of MRI parameters, demographics and risk factors for participants who dropped out during the study compared against those who continued

**Final time point**	**Time 1**		**Time 2**	
**Outcome**	**Withdrew**	**Continued**	**Withdrew**	**Continued**
***n***	**18**	**81**	**10**	**71**
**Demographics and risk factors**				
Age at time point (years)	68.93 (12.10)	69.97 (10.01)	74.05 (11.41)	70.71 (9.25)
Female ( *n* )	5	28	2	26
Male ( *n* )	13	53	8	45
Hypertension ( *n* )	17	75	9	66
Hypercholesterolaemia ( *n* )	16	69	7	62
Current smoker ( *n* )	4	17	2	15
Ex-smoker ( *n* )	8	26	4	22
Diabetes ( *n* )	0	19	2	17
**Volume measurements**				
Mean grey matter (mm ^3^ )	655 534 (61 751)	656 034 (66 053)	644 884 (64 900)	652 157 (66 547)
Mean white matter (mm ^3^ )	307 950 (64 825)	327 490 (58 089)	312 384 (45 257)	330 778 (56 376)
Mean total cerebral volume (mm ^3^ )	1 022 801 (104 170)	1 025 065 (103 547)	1 017 806 (69 871)	1 020 077 (105 874)
Median WMH (mm ^3^ )	47 135 ^**^ (35 834)	**35 370 (29 271)**	53 975 ^***^ (32 848)	**31 111 (25 314)**
Median lacunar infarct (mm ^3^ )	120 (637)	279 (757)	332 (931)	294 (770)
**Percentage change from baseline (%)**				
Mean grey matter	−4.50 (2.21)	−4.02 (2.15)	−4.26 (3.10)	−3.80 (1.67)
Mean white matter	−3.00 (3.07)	−2.44 (4.45)	−6.23 ^***^ (6.72)	**−1.5 (3.32)**
Mean total cerebral volume	−2.36 (1.41)	−2.46 (1.23)	−3.15 (1.16)	−3.22 (6.20)
Median WMH	21.14 (37.77)	+13.34 (31.97)	+12.72 (434)	+16.21 (693)
Median lacunar infarct	+31.10 (35.19)	+19.61 (12.85)	+11.19 (32.97)	+13.95 (30.22)
** Annualized fitted rates (mm ^3^ /year) **				
Mean grey matter	−30 373 (15 488)	−25 647 (13 692)	−14 202 (10 222)	−14 469 (13 851)
Mean white matter	−9273 (9060)	−7845 (12 973)	−10 405 (12 355)	−6406 (15 973)
Mean total cerebral volume	−24 598 (12 234)	−24 958 (12 234)	−15 805 (5604)	−15 522 (28 293)
Median WMH	+12 914* (15 317)	**+5860 (9043)**	+8892* (4127)	**+4825 (5453)**
Median lacune	+39 (133)	+29 (133)	+28 (84)	+26 (135)

Values in brackets are SD, * *P* < 0.05, ^**^P < 0.005, ^***^P < 0.0005.

## Discussion

This study has characterized the longitudinal progression of WMH in symptomatic cerebral small vessel disease. We have shown changes in normal and lesioned tissue subtypes both in terms of absolute volumes and fitted rates, and demonstrated an association particularly between the grey matter and WMH volumes and rates of change. Our results suggest that WMH growth is linked to the underlying white matter anatomy of the long association fasciculi. Furthermore, we have shown that the rate of WMH growth is strongly correlated with regional grey matter atrophy, and that grey matter atrophy contributes most to the secondary reductions in global brain volume that have been previously observed. The results also suggest a phenotypic specific pattern in the rate of grey matter loss associated with small vessel disease severity that is characterized by increasing fronto-parieto-occipital atrophy with notable sparing of the medial temporal lobe and hippocampus. Taken together, these observations suggest that the rate of WMH growth is due to disruption of the end arterial supply from the long perforators as opposed to more proximal occlusion, and that this damage is related to regional cortical atrophy through secondary downstream denervation (‘disconnection phenomenon’).

### Longitudinal white matter hyperintensity progression

The MRI appearance of WMH lesions can arise from a diverse range of pathological events ( Pantoni and Garcia, 1997 ). In cerebral small vessel disease, myelin rarefaction with axonal destruction is found particularly in the more diffuse centrum semiovale lesions ( Revesz *et al.* , 1989 ) that are associated with hyalinization of the long perforating arterioles impairing the axonal vascular supply ( Erten-Lyons *et al.* , 2013 ) and upregulation of endothelial hypoxic markers ( Fernando *et al.* , 2006 ). These form watershed zones where the white matter is particularly vulnerable to damage ( DeCarli *et al.* , 2005 ). In our study, the uniform high frequency of observed WMH occurrence within the deep non-gyral white matter that crosses vascular territories would be consistent with this proposed long perforator pathology. It has been suggested that cortical microinfarcts may also contribute to WMH formation ( Smith *et al.* , 2012 ). However, this current work demonstrates structured heterogeneity in the rate of progression WMH lesions that aligns with underlying myeloarchitecture ( Fig. 3 ). We have demonstrated that WMH progress more rapidly when located within regions of long association fasciculi compared to other white matter regions; however, once lesions are present within these structures they progress at a relatively uniform rate. This suggests that there is underlying variation in the axonal vulnerability to ischaemic damage. The observation of a highly structured yet uniform pattern would be extremely unlikely if a more randomly distributed process, such as focal cortical micro-infarcts or subcortical lacunes ( Supplementary Fig. 3 ), were contributing significantly to the rate of WMH formation, or alternatively if WMH expanded uniformly from the distal edge of pre-existing lesions. These long fascicles are generally densely packed and highly myelinated ( Curnes *et al.* , 1988 ) and critically dependent on a continuous supply of oxygen and glucose mediated by astrocytes ( Pantoni *et al.* , 1996 ; Wender *et al.* , 2000 ; Cambron *et al.* , 2012 ) to support their metabolic demand. The observed vulnerability may emerge through a combination of their remote location from cortical mantle, with subsequent dependence on end arterial supply from the long perforators, which are prone to vascular damage and hyalinization, combined with a higher metabolic demand due to the intrinsic myeloarchitectural properties within the fascicles. This two-hit hypothesis would also account for the temporal fascicles (inferior longitudinal fascicius, uncinate fasciculus) being sparsely affected in age-related cerebral small vessel disease. However, while this may partly account for the observed maps of WMH progression, it does not fully explain the more severe involvement that is apparent within the corticospinal tract, that has also previously been observed in CADASIL (cerebral autosomal-dominant arteriopathy with subcortical infarcts and leukoencephalopathy; Craggs *et al.* , 2014 ). Further work is required to better explain these observations, both to clarify and map white matter metabolism *in vivo* and to quantify regional axonal and myelin densities in humans.

### Rate of grey matter atrophy and functional implications

Previous work demonstrated cortical thinning associated with WMH volume ( Lambert *et al.* , 2015 ; Tuladhar *et al.* , 2015 ), and proposed a phenotypic pattern of cortical atrophy that characterizes increasing WMH severity independently of age, namely parieto-occipital, posterio-superior temporal, cingulate, middle frontal, orbitofrontal cortical thinning, with the previously observed sparing of the medial temporal lobe and hippocampus ( Du *et al.* , 2005 ). This study builds upon those cross-sectional observations by demonstrating an increasing rate of atrophy in these regions as the rate of WMH growth increases, while controlling for age and lacunar infarct ( Appelman *et al.* , 2009 ), and that it is this regional grey matter decline that contributes most to global brain atrophy measures rather than volumetric white matter loss. The observation that the majority of volume loss occurs in the cortex, quite remote from the regions of WMH growth, means that mis-segmentation of the T _1_ images due to WMH is unlikely to account for this result. Furthermore, this study has taken great lengths to minimize any issues relating to tissue mis-segmentation. Functionally these cortical regions overlap, particularly in the frontal areas, with previously described grey matter changes in small vessel disease associated with gait ( de Laat *et al.* , 2012 ) and disturbances in executive function and processing speed ( Gunning-Dixon and Raz, 2003 ; Raji *et al.* , 2012 ). This pattern of atrophy, coupled with the strong association with WMH load and regional progression, would be consistent with a white matter disconnection phenomenon and is concordant with the previously described changes in cortical networks in small vessel disease ( Lawrence *et al.* , 2014 ) and in CADASIL ( Duering *et al.* , 2011 ). These observations suggest that therapies to slow or halt end-arteriole damage and therefore reduce WMH progression would slow the rate of cortical atrophy due to small vessel disease, preventing the secondary functional morbidity that has been well described with these changes ( Nitkunan *et al.* , 2011 ; de Laat *et al.* , 2012 ; Jokinen *et al.* , 2012 ).

### Limitations

There are several limitations with this current work. First, while every effort was made to develop a fully automated, non-biased approach, the segmentations still required a degree of manual correction. However, better quality isotropic data using higher field strengths and image resolutions resulting in improved tissue contrast would be required to refine this approach further to allow a fully automated, non-biased pipeline that would enable increasingly accurate quantification of disease. Additionally, the lacune correction method used will only provide an approximation of the expected tissue type. This could be potentially improved in the future by using a more elaborate model, for example using large datasets of normal brain MRI scans to train a classifier to estimate a missing voxel information (due to an ischaemic lesion) based upon surrounding neighbourhood information.

As described in the ‘Introduction’ section, small vessel disease ranges from mild asymptomatic disease to severe disease with dementia. To allow study of a more homogenous group we applied strict inclusion criteria of a symptomatic lacunar infarct as well as confluent leukoaraiosis. Nevertheless, there was some heterogeneity within our population as suggested by the extreme outliers, whose rate of disease progression was an order of magnitude higher than the rest of the group. Additionally, the dropout cohort had more rapidly progressive small vessel disease, as indicated by the volumes and rates of progression of WMH compared to those who continued in the study ( Table 3 ). A *post hoc* subgroup analysis of the MRI parameters according to participant endpoint ( Supplementary Table 1 ) indicates that those who converted to dementia, died, or had a major stroke had higher overall rates of WMH lesion load and annualized progression compared to those who voluntarily withdrew from the study. However, there is not sufficient data for formal analysis and further work with larger numbers of subjects is required to explore these relationships. While the overall number of subjects may be a limitation in this regard, in relation to all previous longitudinal studies examining WMH progression using MRI ( Supplementary Table 2 ), our work is comparable [all previous studies: median number of subjects = 119 (range 12–1919), average delay = 3.66 years, average number of time points = 2], and sufficient to detect widespread, statistically significant anatomical changes associated with WMH progression which was the primary objective of this work. Additionally, it should also be acknowledged that the cohort of ‘symptomatic small vessel disease’ characterized by this work may not necessarily be representative of same pathology observed in sporadic ‘age-related small vessel disease’, and the results should be interpreted accordingly.

Although our work suggest an important role for WMH in the progression of brain atrophy it cannot completely exclude additional contributions from other processes, such as cortical microinfarcts or denervation secondary to subcortical lacunes. While we have attempted to control for overall lacunar damage by using the total volume of tissue damage as a covariate in our analysis, this fails to account for local downstream lacune effects ( Duering *et al.* , 2015 ). However, for lacunar damage to significantly change the focal rates of atrophy and survive family wise error correction in a voxel-wise analysis, new regions of lacunar damage would need to either overlap or cluster closely over the majority of subjects. Instead we found that the spatial distribution of new lacune damage is widely spread, with preponderance for the striatum but low levels of overlap across subjects ( Supplementary Fig. 3 ). Furthermore, by sampling the volume of grey matter loss within significant regions ( Fig. 4 ), we estimate that the amount of atrophy observed in our study is ∼20 times larger than that reported by Duering *et al.* (2015) . In summary, it is unlikely that lacunar damage contributes significantly to the regional variation observed, both in rates of atrophy and WMH growth.

Additionally, a lack of a control group means that normal ageing effects on this process cannot be entirely excluded. However, the multiple regression model included age as a confounding covariate, therefore is unlikely to contribute significantly to the associations between WMH and grey matter rates. Additionally, we found no significant correlations between grey matter rates, WMH rates and age, and also the overall WMH volume did not correlate with age or total cerebral volume at baseline in our cohort. This means multicollinearity problems ( Kraha *et al.* , 2011 ; Lambert *et al.* , 2015 ) due to relationships between age and WMH are unlikely to be a significant issue in this work.

While we suggest the pattern of grey matter atrophy described may represent a so-called ‘phenotypic pattern’ for small vessel disease, this is by no means definitive as it is based on qualitative comparisons between earlier results reported by ourselves ( Lambert *et al.* , 2015 ) and Tuladhar *et al.* (2015) , being compared against previous published findings in ageing ( Draganski *et al.* , 2011 ; Tamnes *et al.* , 2013 ), dementia ( Whitwell *et al.* , 2007 ; Ossenkoppele *et al.* , 2015 ) and parkinsonian syndromes ( Price *et al.* , 2004 ; Tessa *et al.* , 2014 ). Further work would be required to directly compare these and ascertain the contributions of each to spatial patterns of cortical atrophy, both occurring in isolation and in combination with one another.

Finally, while we acknowledge that this current work not report any neuropsychological correlates, each subject is still being followed-up clinically with an annual neuropsychological battery ( Lawrence *et al.* , 2013 ). Over the 3-year period reported here, there was no marked decline in the measured cognitive parameters and therefore we lacked the power to look for neuroanatomical correlates. However, we are following cases up for 5 years. In subjects who have reached this time point there now appears to be a significant decline in cognition, and future work will analyse this formally once the follow-up period has been fully completed.

## Conclusion

In conclusion this work further defines the pattern of WMH growth in symptomatic cerebral small vessel disease, and demonstrated that the rate of WMH growth is linked to the underlying white matter anatomy of the long association fasciculi. Additionally, we have shown that the rate of WMH growth is strongly correlated with regional grey matter atrophy, which in turn causes the secondary reductions in global brain volume, that have been previously observed to be related to other cognitive and functional outcomes. It suggests an important role of WMH progression in the genesis of brain atrophy and implies that therapies aimed at reducing WMH progression via end-arteriole damage may also protect against secondary brain atrophy and consequent functional morbidity. The magnetic resonance-based morphometric techniques developed here provide 3D, sensitive, quantitative metrics of small vessel disease severity and progression that can be used in the future for patient stratification and objectively monitoring therapeutic clinical trials in this highly prevalent disease.

## Supplementary Material

Supplementary Data

Supplementary Fig. 1

## Abbreviations

CADASIL: cerebral autosomal-dominant arteriopathy with subcortical infarcts and leukoencephalopathy
FLAIR: fluid-attenuated inversion recovery
SPM: Statistical Parametric Mapping
TPM: Tissue Probability Map
WMH: white matter hyperintensities
