# Supplementary material for: Longitudinal patterns of leukoaraiosis and brain atrophy in symptomatic small vessel disease
Source: Brain. 2016 Mar 1;139(4):1136–51. doi: 10.1093/brain/aww009 (PMC4806220; doi:10.1093/brain/aww009)
Supplement: Supplementary Data [file aww009_supplementary_data.zip › brain-2015-01180-File010.pdf]

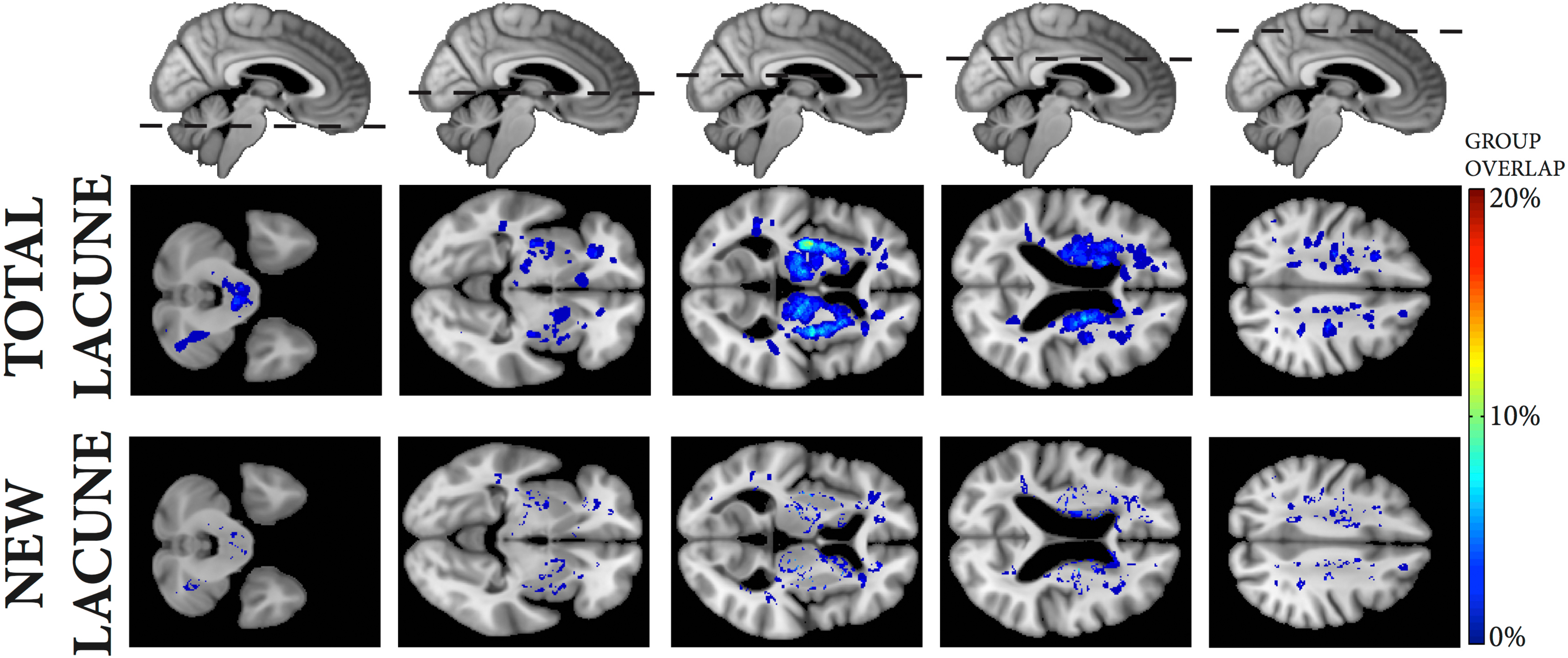

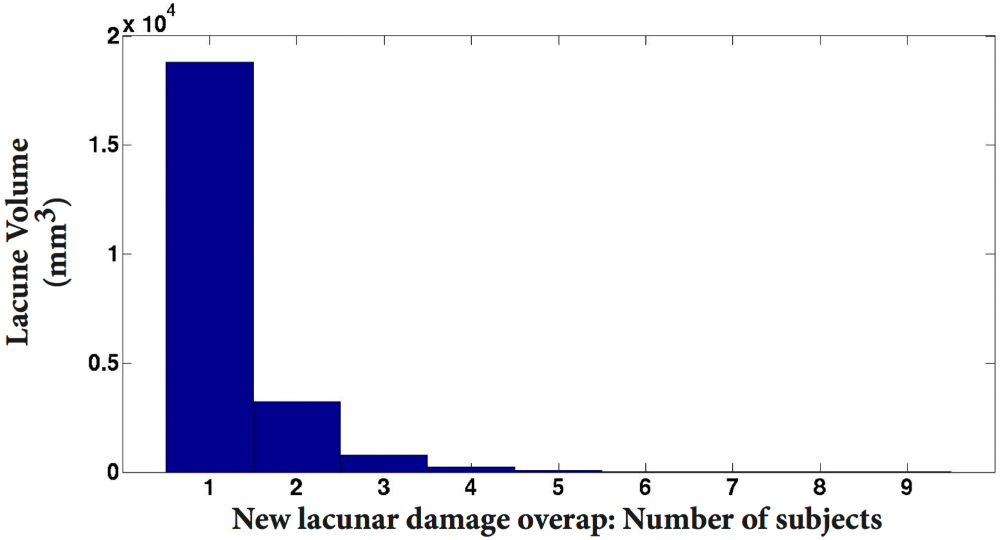

Group-level lacune overlap: Number of subjects	Absolute volume of new lacune tissue (mm^3)	Cumulative volume of new lacune tissue (mm^3)
9	2	2
8	2	4
7	10	14
6	29	43
5	74	117
4	241	358
3	772	1130
2	3251	4381
1	18806	23187
