# Supplementary material for: Longitudinal patterns of leukoaraiosis and brain atrophy in symptomatic small vessel disease
Source: Brain. 2016 Mar 1;139(4):1136–51. doi: 10.1093/brain/aww009 (PMC4806220; doi:10.1093/brain/aww009)
Supplement: Supplementary Data [file aww009_supplementary_data.zip › brain-2015-01180-File011.pdf]

		VOLUME	*RATE PER YEAR*
**OUTCOME**	***n***	**MEDIAN WMH (mm^3)**	**ANNUALISED WMH RATE (mm^3)**
**DECEASED**	6	54644 (*35385*)	+14117 (*9816*)
**DEMENTIA**	3	55184 (*49977*)	+17250 (*28783*)
**MAJOR STROKE**	1	122699 (*13541*)	+13025 (*0*)
**CARDIAC ARREST**	1	36963 (*2737*)	+2656 (*0*)
**DECLINED**	5	47143 (*30086*)	+10938 (*6029*)
**WITHDREW**	5	47127 (*22762*)	+9100 (*9418*)
**LOST**	7	63537 (*33092*)	+7811 (*6516*)
