# Supplementary material for: Longitudinal patterns of leukoaraiosis and brain atrophy in symptomatic small vessel disease
Source: Brain. 2016 Mar 1;139(4):1136–51. doi: 10.1093/brain/aww009 (PMC4806220; doi:10.1093/brain/aww009)
Supplement: Supplementary Data [file aww009_supplementary_data.zip › brain-2015-01180-File012.pdf]

Supplementary Table 2

Publication	Study Population	*n*	Mean Age (Y)	Delay (Y)	Time-points
Veldink et al., 1998	Normal Ageing	14	76	2	3
Whitman et al., 2001	Normal Ageing & gait	70	79	4	2
Yamauchi et al., 2002	Symptomatic Lacune & Normal with Dizziness or Headache	89	66	1.5	2
Nebes et al., 2002	Normal Ageing & depression (control subjects cited)	12	73.5	3	2
Taylor et al., 2003	Normal Ageing	117	69.1	2	2
Schmidt et al., 2003	Normal Ageing	292	60.2	3	2
	Normal Ageing	243		6	3
Dufouil et al., 2005	Cerebrovascular Disease	192	60.7	3	2
Garde et al., 2005	Normal Ageing	26 (698)	80.7	3.8	2
Longstreth et al., 2005	Normal Ageing	1919	74.1	5	2
Mungas et al., 2005	Cognitively mixed. Normal control group cited.	58	74.1	5.6	2
Holtmannspötter et al., 2005	CADASIL	62	44.9	2.2	2
van den Heuvel et al., 2006	Normal Ageing	554	75	3	2
	Normal Ageing				
Burton et al., 2006	Cognitively mixed. Control group cited.	83	74.4	1	2
Chen et al., 2006	Cerebral amyloid angiopathy	26	69.1	1.1	2
Kramer et al., 2007	Normal Ageing	50	73.9	3.75	2
Steffans et al., 2007	Older Depressed Subjects	161	69.23	2	2
Kraut et al., 2008	Normal Ageing	74	70	9	2
van Dijk et al., 2008	Normal Ageing	668	71	3	2
Gouw et al., 2008	Subcortical Iscaemic Vascular Dementia	394	73.1	3	2
Silbert et al., 2009	Normal Ageing	49 (98)	84.1	10.7	3
Goos et al., 2010	Memory Clinic Population	204	66	2	2
Perrin et al., 2010	Chronic Fatigue Syndrome	27	35.8	1	2
Carmichael et al., 2010	Cognitively mixed. Total (and control) groups cited.	804 (224)	76 (76)	1	3
Debette et al., 2011	Normal Ageing (WMH rate from supplementary	1352 (1493)	61	6	2

	data)				
Umemura et al., 2011	Type 2 diabetes mellitus	190	62.7	6	3
Godin et al., 2011	Normal Ageing	1319	72.4	4	2
Knopman et al., 2011	Atherosclerosis Risk	1112	61.7	10.6	2
Maillard et al., 2012	Normal Ageing	150	74.7	4	2
Raz et al., 2012	Normal Ageing	37 (40)	63.3	2.5	3
Grimmer et al., 2012	Probable AD	22	62.7	2.3	2
Sigurdsson et al., 2012	Normal Ageing	367	76	2.5	2
Moscufo et al., 2012	Normal Ageing	77	82	2	2
Wolfson et al., 2013	Normal Ageing	99	81.7	4	3
Maillard et al., 2013	Cognitively mixed. Total (and control) groups cited.	119 (72)	74.5 (74.2)	3.7 (4.1)	2
Liu et al., 2013	Normal Ageing	185	77.47	2	2
Kloppenborg et al., 2014	Symptomatic atherosclerotic disease	663	59 [4]	3.9	2
Erdélyi-Bótor et al., 2014	Migraine	17	47	3	2
Brickman et al., 2015	Normal Ageing (total cited)	303	79.24	4.61	2

Literature Review

To obtain a representative sample of the literature, a Pubmed search was performed using the terms “*longitudinal “small vessel disease’*”, “*longitudinal SVD*”, “*longitudinal leukoaraiosis*”, “*longitudinal leucoaraiosis*”, “*longitudinal “white matter hyperintensities’*”, “*longitudinal “white matter hyperintensity’*”, and “*longitudinal WMH*”. This initially identified a total of 486 publications. Any replicates were removed, and then were further refined by excluding review articles, non-human studies, cohorts with inflammatory pathology, or studies that did not use structural MRI. These remaining were manually reviewed, and only those that quantified the rate or amount of WMH change as a discrete metric, derived from two or more MRI scans over a time period greater than one month, were included. In some circumstances, there were multiple publications from the same study cohorts. Where these were clearly identifiable, only a single representative publication was selected. A final total of 40 publications were reviewed.

References:

Brickman, A. M., Zahodne, L. B., Guzman, V. A., Narkhede, A., Meier, I. B., Griffith, E. Y., Provenzano, F.A., Schupf, N., Manly,J.J., Stern, Y., Luchsinger, J.A. & Mayeux, R. (2014). Reconsidering harbingers of dementia: progression of parietal lobe white matter hyperintensities predicts Alzheimer's disease incidence. *Neurobiology of aging*.

Burton, E. J., McKeith, I. G., Burn, D. J., Firbank, M. J., & O'Brien, J. T. (2006). Progression of white matter hyperintensities in Alzheimer disease, dementia with Lewy bodies, and Parkinson disease dementia: a comparison with normal aging. *The American journal of geriatric psychiatry*, *14*(10), 842-849.

Carmichael, O., Schwarz, C., Drucker, D., Fletcher, E., Harvey, D., Beckett, L., Jack C,R., Weiner M., & DeCarli, C. (2010). Longitudinal changes in white matter disease and cognition in the first year of the Alzheimer disease neuroimaging initiative. *Archives of neurology*, *67*(11), 1370-1378.

Chen, Y. W., Gurol, M. E., Rosand, J., Viswanathan, A., Rakich, S. M., Groover, T. R., ... & Smith, E. E. (2006). Progression of white matter lesions and hemorrhages in cerebral amyloid angiopathy. *Neurology*, *67*(1), 83-87.

Debette, S., Seshadri, S., Beiser, A., Au, R., Himali, J. J., Palumbo, C., Wolf, P.A. & DeCarli, C. (2011). Midlife vascular risk factor exposure accelerates structural brain aging and cognitive decline. *Neurology*, *77*(5), 461-468.

Dufouil, C., Chalmers, J., Coskun, O., Besançon, V., Bousser, M.G., Guillon, P., MacMahon, S., Mazoyer, B., Neal, B., Woodward, M., Tzourio-Mazoyer, N., Tzourio, C. & PROGRESS MRI Substudy Investigators, 2005, Effects of blood pressure lowering on cerebral white matter hyperintensities in patients with stroke: the PROGRESS (Perindopril Protection Against Recurrent Stroke Study) Magnetic Resonance Imaging Substudy, *Circulation*, 112(11), pp. 1644-50.

Erdélyi-Bótor, S., Aradi, M., Kamson, D. O., Kovács, N., Perlaki, G., Orsi, G., ... & Pfund, Z. (2015). Changes of Migraine-Related White Matter Hyperintensities After 3 Years: A Longitudinal MRI Study. *Headache: The Journal of Head and Face Pain*, *55*(1), 55-70.

Garde, E., Lykke Mortensen, E., Rostrup, E. & Paulson, O.B., 2005, Decline in intelligence is associated with progression in white matter hyperintensity volume, *Journal of neurology, neurosurgery, and psychiatry*, 76(9), pp. 1289-91.

Godin O, Tzourio C, Maillard P, et al., Antihypertensive treatment and change in blood pressure are associated with the progression of white matter lesion volumes: the three-city (3C)-dijon magnetic resonance imaging study, *Circulation*, 2011;123:266–73

Goos, J. D. C., Henneman, W. J. P., Sluimer, J. D., Vrenken, H., Sluimer, I. C., Barkhof, F., Blankenstein, B.H., Scheltens, P.H. & Van Der Flier, W. M. (2010).

Incidence of cerebral microbleeds A longitudinal study in a memory clinic population. *Neurology*, *74*(24), 1954-1960.

Gouw, A.A., van der Flier, W.M., Fazekas, F., van Straaten, E.C., Pantoni, L., Poggesi, A., Inzitari, D., Erkinjuntti, T., Wahlund, L.O., Waldemar, G., Schmidt, R., Scheltens, P., Barkhof, F. & LADIS Study Group, 2008, Progression of white matter hyperintensities and incidence of new lacunes over a 3-year period: the Leukoaraiosis and Disability study, *Stroke; a journal of cerebral circulation*, 39(5), pp. 1414-20.

Grimmer, T., Faust, M., Auer, F., Alexopoulos, P., Förstl, H., Henriksen, G., Perneczky, R., Sorg, C., Yousefi, B.H., Drzezga, A. & Kurz, A. (2012). White matter hyperintensities predict amyloid increase in Alzheimer's disease. *Neurobiology of aging*, *33*(12), 2766-2773.

Holtmannspotter M, Peters N, Opherk C, Martin D, Herzog J, Bruckmann H, Samann P, Gschwendtner A, Dichgans M. 2005, Diffusion magnetic resonance histograms as a surrogate marker and predictor of disease progression in CADASIL: a two-year follow-up study. 36:2559–2565

Kloppenborg, R. P., Geerlings, M. I., Visseren, F. L., Mali, W. P., Vermeulen, M., van der Graaf, Y., & Nederkoorn, P. J. (2014). Homocysteine and progression of generalized small-vessel disease The SMART-MR Study. *Neurology*, *82*(9), 777-783.

Knopman, D. S., Penman, A. D., Catellier, D. J., Coker, L. H., Shibata, D. K., Sharrett, A. R., & Mosley, T. H. (2011). Vascular risk factors and longitudinal changes on brain MRI The ARIC study. *Neurology*, *76*(22), 1879-1885.

Kramer JH, Mungas D, Reed BR, et al. 2007. Longitudinal MRI and cognitive change in healthy elderly. Neuropsychology 21: 412– 418.

Kraut, M.A., Beason-Held, L.L., Elkins, W.D. & Resnick, S.M., 2008, The impact of magnetic resonance imaging-detected white matter hyperintensities on longitudinal changes in regional cerebral blood flow, *Journal of cerebral blood flow and metabolism : official journal of the International Society of Cerebral Blood Flow and Metabolism*, 28(1), pp. 190-7.

Liu, T., Sachdev, P. S., Lipnicki, D. M., Jiang, J., Geng, G., Zhu, W., Reppermund, S., Tao, D., Trollor, J.N., Brodaty, H. & Wen, W. (2013). Limited relationships between two-year changes in sulcal morphology and other common neuroimaging indices in the elderly. *NeuroImage*, *83*, 12-17.

Longstreth, W. T., Arnold, A. M., Beauchamp, N. J., Manolio, T. A., Lefkowitz, D., Jungreis, C., Hirsch, C. H., O'Leary D. H., & Furberg, C. D. (2005). Incidence, Manifestations, and Predictors of Worsening White Matter on Serial Cranial Magnetic Resonance Imaging in the Elderly The Cardiovascular Health Study. *Stroke*, *36*(1), 56-61.

Maillard, P., Carmichael, O., Fletcher, E., Reed, B., Mungas, D., & DeCarli, C. (2012). Coevolution of white matter hyperintensities and cognition in the elderly. *Neurology*, *79*(5), 442-448.

Maillard, P., Carmichael, O., Harvey, D., Fletcher, E., Reed, B., Mungas, D., & DeCarli, C. (2013). FLAIR and diffusion MRI signals are independent predictors of white matter hyperintensities. *American Journal of Neuroradiology*, *34*(1), 54-61.

Moscufo, N., Wolfson, L., Meier, D., Liguori, M., Hildenbrand, P. G., Wakefield, D., Schmidt, J.A., Pearlson, G.D., & Guttmann, C. R. (2012). Mobility decline in the elderly relates to lesion accrual in the splenium of the corpus callosum. *Age*, *34*(2), 405-414.

Mungas, D., Harvey, D., Reed, B.R., Jagust, W.J., DeCarli, C., Beckett, L., Mack, W.J., Kramer, J.H., Weiner, M.W., Schuff, N. & Chui, H.C., 2005, Longitudinal volumetric MRI change and rate of cognitive decline, *Neurology*, 65(4), pp. 565-71.

Nebes, R. D., Reynolds, C. F., Boada, F., Meltzer, C. C., Fukui, M. B., Saxton, J., Halligan, E.M. & DeKosky, S. T. (2002). Longitudinal increase in the volume of white matter hyperintensities in late-onset depression. *International journal of geriatric psychiatry*, *17*(6), 526-530.

Perrin, R., Embleton, K., Pentreath, V. W., & Jackson, A. (2010). Longitudinal MRI shows no cerebral abnormality in chronic fatigue syndrome. The British Journal of Radiology, 83(989), 419–423

Raz, N., Yang, Y.Q., Rodrigue, K.M., Kennedy, K.M., Lindenberger, U. & Ghisletta, P., 2012, White matter deterioration in 15 months: latent growth curve models in healthy adults, *Neurobiology of aging*, 33(2), pp. 429.e1-5.

Schmidt, R., Enzinger, C., Ropele, S., Schmidt, H. & Fazekas, F., 2003, Progression of cerebral white matter lesions: 6-year results of the Austrian Stroke Prevention Study, *The Lancet*, 361(9374), pp. 2046-8.

Sigurdsson, S., Aspelund, T., Forsberg, L., Fredriksson, J., Kjartansson, O., Oskarsdottir, B., Jonssona, P.V., Eiriksdottir, G., Harris, T.B., Zijdenbos, A., van Buchem, M.A., Launer, L.J. & Gudnason, V. (2012). Brain tissue volumes in the general population of the elderly: the AGES-Reykjavik study. *Neuroimage*, *59*(4), 3862-3870.

Silbert, L. C., Howieson, D. B., Dodge, H., & Kaye, J. A. (2009). Cognitive impairment risk White matter hyperintensity progression matters. *Neurology*, *73*(2), 120-125

Steffens, D.C., Potter, G.G., McQuoid, D.R., MacFall, J.R., Payne, M.E., Burke, J.R., Plassman, B.L. & Welsh-Bohmer, K.A., 2007, Longitudinal magnetic resonance imaging vascular changes, apolipoprotein E genotype, and development of dementia in the neurocognitive outcomes of depression in the elderly study, *The American journal of geriatric psychiatry : official journal of the American Association for Geriatric Psychiatry*, 15(10), pp. 839-49.

Taylor, W.D., MacFall, J.R., Provenzale, J.M., Payne, M.E., McQuoid, D.R., Steffens, D.C. & Krishnan, K.R.R., 2003, Serial MR imaging of volumes of

hyperintense white matter lesions in elderly patients: correlation with vascular risk factors, *American Journal of Roentgenology*, 181(2), pp. 571-6.

Umemura, T., T. Kawamura, H. Umegaki, S. Mashita, A. Kanai, T. Sakakibara, N. Hotta, and G. Sobue. "Endothelial and inflammatory markers in relation to progression of ischaemic cerebral small-vessel disease and cognitive impairment: a 6-year longitudinal study in patients with type 2 diabetes mellitus." *Journal of Neurology, Neurosurgery & Psychiatry* (2011)

Van den Heuvel, D.M.J., Ten Dam, V.H., de Craen, A.J.M., Admiraal-Behloul, F., Olofsen, H., Bollen, E.L.E.M., Jolles J., Murray HM., Blauw G.J., Westendorp W.G.J., & van Buchem, M. A. (2006). Increase in periventricular white matter hyperintensities parallels decline in mental processing speed in a non-demented elderly population. *Journal of Neurology, Neurosurgery & Psychiatry*, *77*(2), 149-153.

van Dijk, E.J., Prins, N.D., Vrooman, H.A., Hofman, A., Koudstaal, P.J. & Breteler, M.M., 2008, Progression of cerebral small vessel disease in relation to risk factors and cognitive consequences: Rotterdam Scan study, *Stroke; a journal of cerebral circulation*, 39(10), pp. 2712-9.

Veldink, J. H., Scheltens, P., Jonker, C., & Launer, L. J. (1998). Progression of cerebral white matter hyperintensities on MRI is related to diastolic blood pressure. *Neurology*, *51*(1), 319-320.

Whitman, G. T., Tang, T., Lin, A., & Baloh, R. W. (2001). A prospective study of cerebral white matter abnormalities in older people with gait dysfunction. *Neurology*, *57*(6), 990-994.

Wolfson, L., Wakefield, D.B., Moscufo, N., Kaplan, R.F., Hall, C.B., Schmidt, J.A., Guttmann, C.R. & White, W.B., 2013, Rapid buildup of brain white matter hyperintensities over 4 years linked to ambulatory blood pressure, mobility, cognition, and depression in old persons, *The journals of gerontology. Series A, Biological sciences and medical sciences*, 68(11), pp. 1387-94.

Yamauchi, H., Fukuda, H. & Oyanagi, C., 2002, Significance of white matter high intensity lesions as a predictor of stroke from arteriolosclerosis, *Journal of Neurology, Neurosurgery & Psychiatry*, 72(5), pp. 576-82.
