# Supplementary material for: Longitudinal patterns of leukoaraiosis and brain atrophy in symptomatic small vessel disease
Source: Brain. 2016 Mar 1;139(4):1136–51. doi: 10.1093/brain/aww009 (PMC4806220; doi:10.1093/brain/aww009)
Supplement: Supplementary Data [file aww009_supplementary_data.zip › brain-2015-01180-File015.pdf]

Supplementary Material

Supplementary material is available:

Supplementary figure 1: MRI space definitions and examples

Supplementary figure 2: Summary of longitudinal MRI volumetric metrics: Showing distribution histogram (column 1), individual trajectories (column 2) and percentage change from baseline (column 3) for grey matter, white matter, total cerebral volume and WMH.

Supplementary figure 3: Summary of longitudinal lacunar damage: Showing the group average overlap in the total distribution of lacune tissue (top), and new regions classified as lacune compared to baseline (bottom). The histogram and table summarize the number of subjects and volume of lacune tissue where overlap occurs at a group average level.

Supplementary table 1: Dropout sub-analysis: Sub-analysis of WMH volume in all participants who withdrew from the study between year one and year three, subdivided according to end point outcome.

Supplementary table 2: Summary of published literature examining longitudinal changes in WMH with MRI.

Supplementary Material: Detailed breakdown of methods.
