# Supplementary material for: Longitudinal patterns of leukoaraiosis and brain atrophy in symptomatic small vessel disease
Source: Brain. 2016 Mar 1;139(4):1136–51. doi: 10.1093/brain/aww009 (PMC4806220; doi:10.1093/brain/aww009)
Supplement: Supplementary Data [file aww009_supplementary_data.zip › brain-2015-01180-File007.pdf]

Methodological Approach - Details

The pipeline constructed (figure 2), whilst optimized for our study population, has been designed to adapt pre-existing free, open source software. Consequently, the technique should be reasonably easy to replicate using T1w and FLAIR images. The dependent step is defining the WMH and lacune lesions, for which there already exists a variety of automated techniques (García-Lorenzo *et al.*, 2013) including free open-source toolboxes (e.g. LST Toolbox (http://www.applied-statistics.de/lst.html), Wisconsin White Matter Hyperintensities Segmentation Toolbox (https://www.nitrc.org/projects/w2mhs/) (Ithapu *et al.*, 2014)) in addition to manual delineation tools (http://www.itksnap.org) (Yushkevich *et al.*, 2006).

Provided below is a more detailed breakdown of each step shown in figure 2 to supplement those provided in the main manuscript, however interested readers are also directed to Lambert *et al.*, 2013 (generation of population specific TPMs using a modified multivariate Mixture of Gaussians) and 2015 (Lesion repair pre-processing pipeline):

- Population Specific TPMs: This was an optional step we adopted based on earlier work (Lambert *et al.*, 2013 & 2015) to try and improve the accuracy of our segmentations within our study population. However, this step is not entirely necessary to replicate the overall technique as the default SPM TPMs can be also used to generate un-repaired GM, WM and CSF. The un-repaired segmentations should be used to generate initial warps to group average space using the *Shoot* toolbox in SPM12 (Ashburner and Friston, 2011), and all tissue segmentations (WM, GM, CSF) warped to that space. Population

average segmentations should be created for each tissue type, so that a voxel-wise probability of a tissue type can be obtained.

- WMH Segmentation: This was specifically developed and optimized for our study population and not yet in a format suitable for general release, though we plan to release it in the future. However, alternative toolboxes for WMH segmentation already exist as detailed above.
- Lacune Segmentation: This was performed manually in ITK-SNAP.
- Lesion repair: Once the segmentations for GM, WM, CSF, WMH and lacunes are available, this step can be implemented in MATLAB. The procedure involves finding all the WMH voxels, and to set the corresponding voxels to zero in GM and one in WM. The lacune repair is an estimation based upon the warped initial GM, WM and CSF population averages. Each lacune is warped to this group average space, and each voxel within this mask is allocated a tissue class based on the highest probability in the group average maps. The "*repaired*" mask is then warped back to individual subject space.
- Warping: All warps were created using the *Shoot* toolbox in SPM12 (Ashburner and Friston, 2011). Initially all the scans for an individual were used to create an average individual subject space. Then these templates for every individual in the group were used to create the group average. This step separately using the repaired and unrepaired segmentations as detailed in the methods and figure 2.

Any additional queries related to this pipeline can be directed to the corresponding author (CL).

References:

Ashburner, J. & Friston, K.J., 2011, Diffeomorphic registration using geodesic shooting and Gauss-Newton optimisation, *NeuroImage*, 55(3), pp. 954-67.

García-Lorenzo, D., Francis, S., Narayanan, S., Arnold, D. L., & Collins, D. L. (2013). Review of automatic segmentation methods of multiple sclerosis white matter lesions on conventional magnetic resonance imaging. *Medical image analysis*, *17*(1), 1-18.

Ithapu, V., Singh, V., Lindner, C., Austin, B. P., Hinrichs, C., Carlsson, C. M., Bendlin, B. B., & Johnson, S. C. (2014). Extracting and summarizing white matter hyperintensities using supervised segmentation methods in Alzheimer's disease risk and aging studies. *Human brain mapping*, *35*(8), 4219-4235.

Lambert, C., Lutti, A., Helms, G., Frackowiak, R. & Ashburner, J., 2013, Multiparametric brainstem segmentation using a modified multivariate mixture of Gaussians, *NeuroImage: Clinical*, 2, pp. 684-94.

Lambert C, Janakan SN, Barrick TR, Markus HS. 2015, Characterising the Grey Matter Correlates of Leukoaraiosis in Cerebral Small Vessel Disease *NeuroImage: Clinical, in press*

Yushkevich, P.A., Piven, J., Hazlett, H.C., Smith, R.G., Ho, S., Gee, J.C. & Gerig, G., 2006, User-guided 3D active contour segmentation of anatomical structures: significantly improved efficiency and reliability, *NeuroImage*, 31(3), pp. 1116-28.
