# Supplementary material for: Longitudinal patterns of leukoaraiosis and brain atrophy in symptomatic small vessel disease
Source: Brain. 2016 Mar 1;139(4):1136–51. doi: 10.1093/brain/aww009 (PMC4806220; doi:10.1093/brain/aww009)
Supplement: Supplementary Data [file aww009_supplementary_data.zip › brain-2015-01180-File008.pdf]

TERM	EXAMPLE	DEFINITION
Native Space	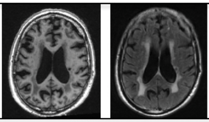	*Structural images that have been aligned with MNI space using affine co-registration, and resliced to 1mm isotropic resolution.*
Individual Longitudinal Dataset	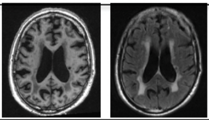	*For each subject (S) there will exist a dataset consisting of between two to four MRI datasets (T_0-T_3) containing aligned, co-registered T1 and FLAIR images in Native Space.*
Unrepaired Segmentation	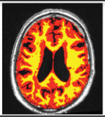	*GM, WM and CSF segmentations from SPM New-Segment where the tissue misclassification caused by WMH and LI has not yet been corrected using the Native Segmentations.*
Native Segmentation	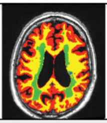	*Using population specific TPMs, these segmentations in native space include automated GM, WM, WMH and CSF tissue classes, as well as regions of LI that have been defined in a semi-automated fashion. These are used for subsequent repair steps.*
Repaired Segmentation	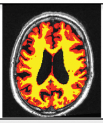	*Native space GM, WM and CSF where the regions of tissue misclassification have been repaired using the native segmentation. These are used for creating repaired templates.*
Unrepaired Individual Average Template	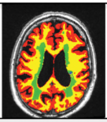	*For each individual longitudinal dataset, the native segmentations (consisting of GM, WM, CSF, WMH and LI) are warped to their individual average template. These are used to calculate rate maps for lesioned tissue i.e. WMH and LI.*
Repaired Individual Average Template	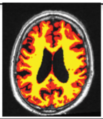	*For each individual longitudinal dataset, the repaired segmentations (GM, WM and CSF) are warped to their repaired individual average template. These are used to calculate rate maps for the main tissue classes i.e. GM, WM and CSF.*
Group Average Repaired Template	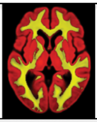	*Using the repaired individual average templates across the entire study population, this is the final group average template where further statistical analysis is performed.*
Rate Maps	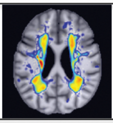	*For each individual longitudinal dataset, a map showing the rate of voxel-wise expansion or contraction. These are generated for the lesioned (WMH and LI) and main (WM, GM and CSF) tissue classes using the unrepaired and repaired individual templates respectively.*
