# Supplementary figures and images for: Longitudinal patterns of leukoaraiosis and brain atrophy in symptomatic small vessel disease
Source: Brain. 2016 Mar 1;139(4):1136–51. doi: 10.1093/brain/aww009 (PMC4806220; doi:10.1093/brain/aww009)

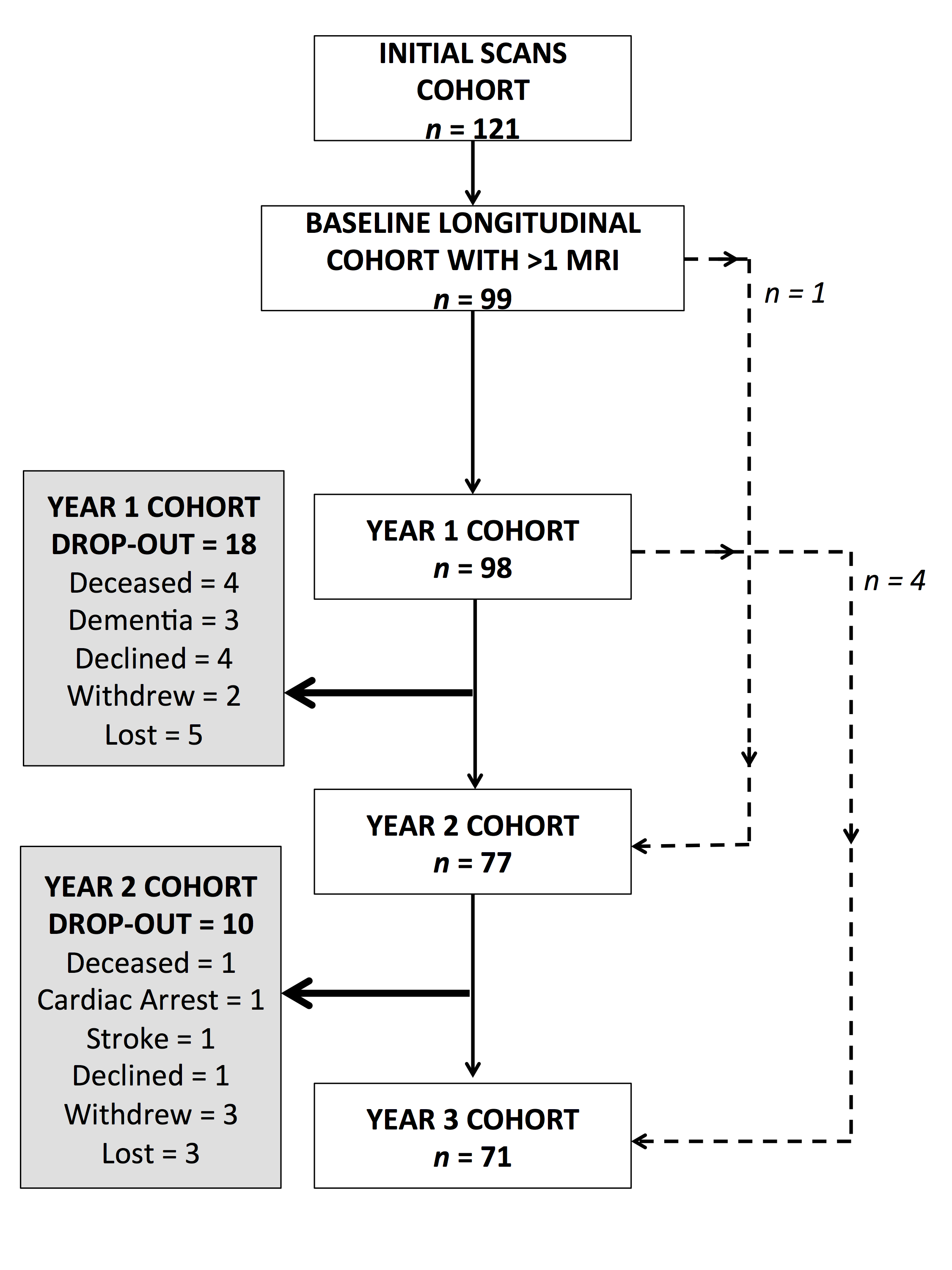

Supplement: Supplementary Data [file aww009_supplementary_data.zip › brain-2015-01180-File014.jpg]
